# Intracellular Chloride Channels: A Rising Target in Lung Disease Research

**DOI:** 10.70322/jrbtm.2025.10012

**Published:** 2025-12-10

**Authors:** Boina Baoyinna Borjigin, Jing Zhao, Harpreet Singh, Yutong Zhao

**Affiliations:** 1 Department of Physiology and Cell Biology, The Davis Heart and Lung Research Institute, The Ohio State University, Columbus, OH 43210, USA;; 2 Department of Internal Medicine, The Ohio State University, Columbus, OH 43210, USA

**Keywords:** CLICs, Chloride channel, Lung cancer, Inflammation, PAH, Signaling pathway

## Abstract

Chloride intracellular ion channels (CLICs) represent a relatively underexplored class of chloride channels and are included in a research initiative that focuses on druggable genes that have not been well studied yet. As a unique family, CLICs exist in membrane and soluble forms and play a role in regulating chloride flux and modulating various aspects of cellular biology. To date, six mammalian CLICs have been cloned and characterized at molecular and physiological levels. The respiratory system, responsible for gas exchange between the atmosphere and the human body, has recently been shown to express CLICs with functional relevance in lung pathophysiology, including lung carcinoma, inflammation, and endothelial dysfunction. Notably, the expression patterns of CLIC isoforms in lung cell types are distinct. Among them, CLIC1, CLIC3, and CLIC4 have been investigated more extensively, particularly in the context of lung cancer, inflammatory diseases, and pulmonary arterial hypertension. A deeper understanding of the role of CLICs in regulating lung cellular function may pave the way for developing novel therapeutic strategies to treat pulmonary disorders. In this review, we summarize the expression and functional roles of CLICs in lung pathophysiology, with particular emphasis on CLIC1, CLIC3, and CLIC4.

## 1. Introduction

Ion channels maintain cellular electrochemical balance and are essential for cellular signaling that governs cell proliferation, migration, death, and inflammatory responses [1–4]. Chloride channels, which are present in all cell types, regulate the movement of chloride ions across membranes. This transport is a passive process dependent on the chloride gradient between intracellular and extracellular concentration or between organelles [5–7]. Beyond its role in maintaining ion homeostasis, fluid balance, and acid-base equilibrium, chloride also acts as a signaling molecule that directly modulates enzyme activity, thereby regulating various cellular functions. For example, alpha-amylases require chloride as an allosteric activator [8], while chloride has been shown to inhibit With-No-Lysine (WNK) kinases [9]. The chloride channel superfamily consists of several subgroups classified by their regulatory mechanism: (1) voltage-gated chloride channels (CLCs), which include nine isoforms (CIC1–7, CIC-ka, and CIC-kb) [10–12]; (2) calcium-activated chloride channels (CaCCs), such as TMEM16A (transmembrane protein 16) and bestrophin 1–4 (BEST1–BEST4) [13,14]; (3) ligand-gated chloride channels, including gamma-aminobutyric acid (GABA) and glycine receptors [15,16]; (4) cystic fibrosis transmembrane conductance regulator (CFTR) which plays a critical role in regulating both chloride and bicarbonate levels in epithelial cells [17–19]; and (5) chloride intracellular ion channels (CLICs), comprising six isoforms (CLIC1–CLIC6) [20–22]. Dysregulation of chloride channels due to inherited mutations links to human disorders. Dominant-negative mutations in the *CICN1* gene, which encodes the CIC-1 chloride channel, are causative of myotonia congenita (Thomsen disease) [23,24]. Genetic mutations in the *CFTR* gene impair CFTR protein stability, disrupt cellular localization, and hinder channel activation, leading to defective fluid homeostasis in epithelial tissues of the lung, pancreas, intestine, and other organs [25–27].

**Figure 1.**
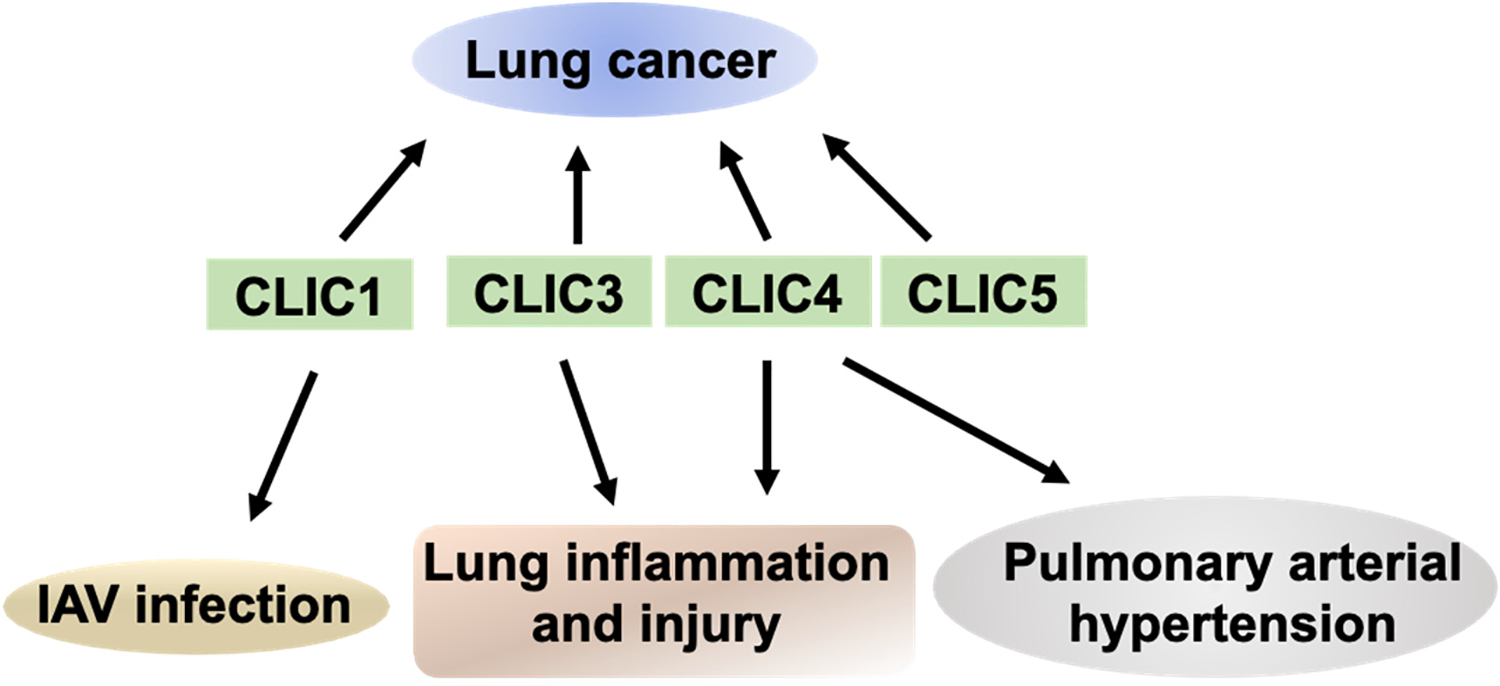
CLICs in lung diseases. CLICs have been implicated in the pathogenesis of various lung diseases, including lung cancer, IAV infection, lung inflammation and injury, and pulmonary arterial hypertension.

The respiratory system supplies the body with oxygen and removes carbon dioxide. In addition to gas exchange, the lungs also play a vital role in maintaining acid-base homeostasis and modulating immune responses. Lung tissue comprises several major cell types, including epithelial, endothelial, fibroblast, and immune cells. Lung diseases often arise from cellular dysfunction or structural abnormalities within lung tissue. The roles of several chloride channels in lung physiology and the development of lung diseases have been investigated. For example, CFTR is a cAMP-activated chloride channel and is highly expressed in lung epithelial cells. The most common CFTR mutation, F508del, causes the protein to be retained in the endoplasmic reticulum, preventing its translocation to the plasma membrane and leading to its rapid degradation by the ubiquitin-proteasome system. Loss of CFTR function in the lung epithelium results in dehydrated and abnormally thick mucus. This abnormal mucus accumulation in the airway obstructs airflow, promotes bacterial growth, and triggers persistent inflammation. Together, these pathological changes ultimately lead to the development of cystic fibrosis [19,26,27]. Another compelling example is the relevance of CaCCs in the pathophysiology of asthma. TMEM16A, a family of CaCCs, is upregulated in airway smooth muscle cells. Increased activation of TMEM16A contributes to the bronchial hyperresponsiveness [28,29]. Extensive studies have demonstrated that abnormal expression of CLICs plays a role in the pathogenesis of certain lung diseases. Elucidating the role of CLICs in regulating lung cellular function may open new avenues for developing innovative therapies to treat pulmonary disorders. This review will focus on summarizing the role of CLICs in lung pathophysiology (Figure 1) and highlighting CLICs as potential targets in treating lung diseases.

## 2. Expression of CLICs in the Lungs

### 2.1. CLIC Family Members and Biophysics Properties

**Figure 2.**
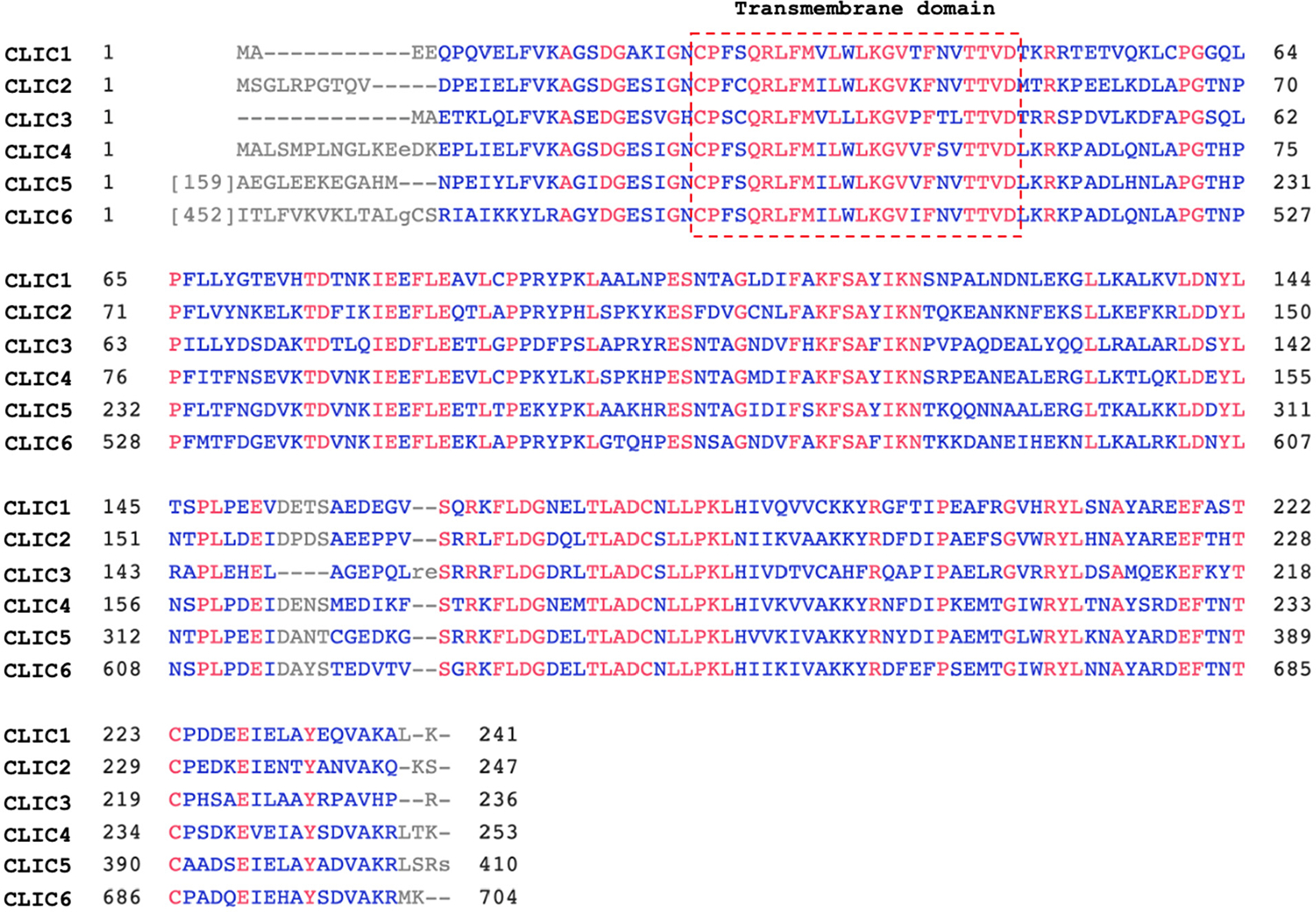
Sequence alignment of human CLIC isoforms. CLIC1–CLIC4 consist of 236–241 amino acids, whereas CLIC5 and CLIC6 exhibit unique N-terminal structural features. Regions of homology and identify within the CLIC activity domain are highlighted in blue and red, respectively. The transmembrane domain is indicated by the red box.

CLICs are a family of chloride channels primarily localized in the cytoplasm as soluble proteins. Soluble CLIC proteins can translocate to the intracellular or plasma membranes under specific conditions, such as changes in pH, ion concentrations, or stimulation by sphingosine-1-phosphate [30,31]. Membrane-bound CLICs form oligomers and function as a channel, and play a role in the regulation of chloride levels. To date, six mammalian homologs have been identified, all sharing a common CLIC structure model [20,21,32] (Figure 2). CLIC5B, also known as p64, was the first CLIC isoform identified in 1993 from bovine kidney [33]. CLIC1–CLIC4 consist of 236–253 amino acid residues, while CLIC5 and CLIC6 (also known as parchorin) possess extended N-terminal domains. Although the structure of CLIC5’s N-terminal remains characterized, the N-terminal domain of CLIC6 shows similarity to the gamma and tau subunits of DNA polymerase III, suggesting that CLIC5 and CLIC6 may exhibit other roles in addition to their chloride channel activation. The conserved structure of CLICs also shows that the soluble CLICs play a role in redox homeostasis, since they share high structural homology with the glutathione S-transferase (GST) fold family [34,35].

### 2.2. CLIC Protein Expression across Lung Cell Types

**Figure 3.**
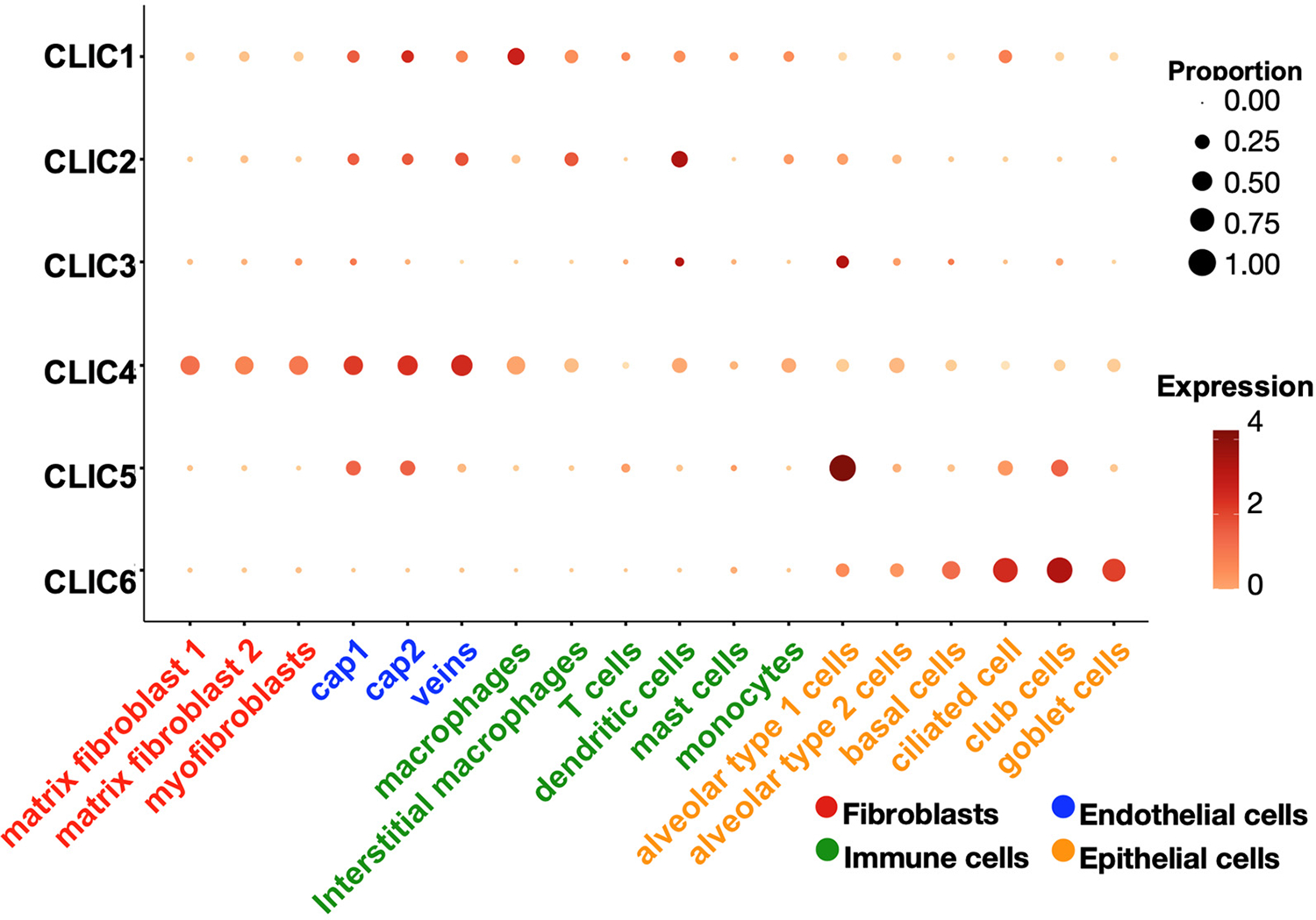
Cellular distribution of CLICs in the lung. CLICs exhibit a distinct expression pattern across various lung cell types, including fibroblasts, epithelial, endothelial, and immune cells. This data was obtained by reanalyzing LungMAP (app.lungmap.net; accessed on 5 October 2025).

All the CLIC isoforms are expressed in the lungs, but their cellular distribution varies in lung cell types. CLIC1, CLIC2, and CLIC4 are broadly expressed across epithelial, endothelial, fibroblast, and immune cells. CLIC3 is also widely expressed, though its levels in macrophages are relatively low. CLIC5 is highly expressed in lung epithelial and endothelial cells, whereas CLIC6 is exclusively found in epithelial cells (Figure 3). These different expression level patterns suggest distinct roles of each CLIC isoform in lung physiology. Epithelial cells, which serve as the first line of defense in the lungs, express all six isoforms. Endothelial cells lining the vasculature express CLIC1, CLIC2, CLIC4, and CLIC5, but not CLIC3 and CLIC6. Fibroblasts, key components of connective tissue, express high levels of CLIC4, with lower levels of other CLICs. Macrophages, essential for innate immune responses in the lungs, express CLIC1, CLIC2, and CLIC4. The finding of the cellular distribution of CLIC isoforms is based on adult human lung cells. The expression of CLIC isoforms in certain cell types may vary during lung development, the progression of lung diseases, and between sexes. For example, CLIC6 shows higher expression in the male heart compared to females [36]. In mouse models, acute alcohol exposure increases CLIC4 expression in the medial prefrontal cortex of female mice, but not in males [37]. However, the sex-based expression patterns of CLIC isoforms in human lung cells have not been reported, indicating a knowledge gap in current research that warrants further investigation. The conserved structural features of CLIC proteins suggest a role in redox regulation, as they share significant structural homology with the glutathione S-transferase (GST) fold family [34,35]. However, the specific involvement of CLIC isoforms in redox reactions related to lung cellular function and pulmonary pathophysiology remains largely unexplored.

## 3. CLIC1 in Lung Diseases

CLIC1 was the first human CLIC identified in 1997, and initially referred to as nuclear chloride channel-27 (NCC27) [38]. It was first detected in the nuclear membrane of CHO cells, but subsequent studies have demonstrated its widespread distribution across various cellular compartments, including intracellular cytoplasmic organelles and the plasma membrane. Its translocation in the cells is regulated by intracellular zinc, pH, and reactive oxidative oxygen species (ROS) [30,39,40].

### 3.1. CLIC1 in Lung Adenocarcinoma

#### 3.1.1. CLIC1 Expression in Lung Adenocarcinoma

Lung adenocarcinoma is the most frequent subtype of non-small cell lung cancer. Although its 5-year survival rate is slightly higher than that of other lung cancer types, overall prognosis remains poor [41]. Yasuda, Y. et al. recruited and analyzed 19 human lung adenocarcinoma subjects with high levels of CLIC1 and 55 subjects with low levels of CLIC1 [42]. The authors demonstrated that CLIC1 expression is significantly correlated with a short overall survival rate and poor prognosis of lung adenocarcinoma [42]. Although the sample size in this study is limited, the finding suggests that CLIC1 may play a vital role in the development of lung carcinoma. The conclusions are based on immunohistochemical analysis of CLIC1 in lung adenocarcinoma tissues. To validate these observations, further studies will involve re-analysis of *CLIC1* gene expression using currently available lung adenocarcinoma datasets.

#### 3.1.2. Molecular Mechanisms of CLIC1 Involvement in Lung Adenocarcinoma

The molecular mechanisms by which CLIC1 regulates lung adenocarcinoma cells have been explored by two research groups. Yasuda, Y. et al. reported that knockdown of CLIC1 via transfection of specific siRNAs led to reduced cell proliferation and migration, along with an enhanced cellular stress response in lung adenocarcinoma cell lines such as A549 and PC9 cells [42]. Additionally, phosphorylation levels of p38 MAPK were decreased in CLIC1-deficient cells [42]. Although the authors proposed that the p38 MAPK signaling pathway mediates these effects, further direct evidence is required to substantiate this conclusion. Lee, J. et al. reported that CLIC1 knockdown exacerbated cellular stress response in A549 cells, as indicated by increased phosphorylation of γH2AX, a key marker of DNA double-strand break repair [43]. In the latter study, the authors demonstrated that CLIC1 knockdown led to elevated intracellular calcium and ROS levels, accompanied by increased phosphorylation of p38 MAPK, JNK, and AKT [43]. Although both studies suggest that CLIC1 plays a role in regulating intracellular signaling pathways, it remains unclear whether these effects are mediated through alterations in intracellular chloride levels. Furthermore, the specific contribution of CLIC1’s function within the cytoplasm versus its activity on the intercellular organelle membrane has yet to be elucidated. Both ROS and calcium serve as signaling mediators that critically regulate lung cancer cell proliferation and survival. Although Lee J. et al. did not directly investigate the role of CLIC1 in cell proliferation, their observation of elevated ROS and calcium levels following CLIC1 knockdown suggests a potential proliferative effect. However, this implication contrasts with findings from Yasuda Y. et al., who reported that CLIC1 knockdown suppresses proliferation, highlighting a discrepancy that warrants further investigation.

### 3.2. CLIC1 in Lung Influenza A Virus Infection

Influenza viruses primarily target the respiratory tract, triggering excessive hyperinflammation and cellular damage, and posing a significant global health threat. Airway epithelial cells form a protective barrier and play a crucial role in host defense by regulating mucus secretion and cilia movement. Gene array analysis revealed an upregulation of *CLIC1* gene expression in differentiated primary bronchial epithelial cells following H1N1 infection [44]. Knockdown of CLIC1 significantly reduced viral replication in A549 cells, consistent with the findings reported by Rashid, M. et al. [45,46]. Collectively, these studies indicate that elevated CLIC1 expression plays a role in the H1N1 infection of lung epithelial cells. Rashid, M., and Coombs, K.M. further investigated the molecular mechanisms by which CLIC1 modulates H1N1 replication in lung epithelial cells. Interestingly, they found that CLIC1 knockdown reduced the release of influenza A virus (IAV) into the cell supernatant, while intracellular viral protein levels remained unchanged, suggesting that CLIC1 plays a role in the later stages of IAV replication [46]. The role of CLIC1 in regulating chloride currents in lung epithelial cells has been reported. CLIC1 knockdown attenuated forskolin-induced chloride currents by 62.7%, while basal chloride currents remained unchanged in human bronchial epithelial cells [47]. Rashid, M., and Coombs, K.M. used the CLIC1 inhibitor to confirm the effect of CLIC1 knockdown in A549 cells, suggesting that the effect of CLIC1 is through its chloride channel activity [46]. Furthermore, proteomic analysis revealed alternations in 149 proteins in CLIC1 knockdown cells, offering new insights into the role of CLIC1 in H1N1 replication [46]. The molecular mechanisms by which IAV infection increases CLIC1 expression and whether CLIC1 modulates IAV infection-mediated inflammatory responses need to be investigated in future studies. A study by Domingo-Fernandez, et al. demonstrated that CLIC1 participates in the regulation of NLRP3 inflammasome [48], suggesting that CLIC1 may modulate host immune responses by regulating NLRP3-inflammasome.

### 3.3. CLIC1 in Cystic Fibrosis

Cystic fibrosis is an inherited genetic disorder caused by mutations in the *CFTR* gene. CLIC proteins regulate chloride levels, suggesting that their dysfunction may contribute to the pathogenesis of cystic fibrosis. Although direct evidence linking CLICs to cystic fibrosis is lacking, an *in vitro* study by Edwards, J. demonstrated that co-expression of CLIC1 and CFTR in Xenopus oocytes increased cAMP-activated whole-cell conductance, an effect reversed by a CLIC inhibitor [49]. Furthermore, the study showed that CFTR activation promoted CLIC1 translocation from the cytoplasm to the membrane [49], indicating a potential role of CLIC1 in cystic fibrosis. However, these findings were obtained using Xenopus oocytes and have not yet been validated in lung airway epithelial cells.

## 4. CLIC3 in Lung Diseases

CLIC3 was first identified in 1999 as a MAPK-associated CLIC, primarily located in the nucleus. The initial study characterized it as a protein that interacts with the MAPK signaling pathway and functions as a chloride channel [50]. The CLIC3 protein functions in a soluble state as a glutaredoxin-like enzyme, catalyzing thiol-disulfide exchange reactions using reduced glutathione. Additionally, CLIC3 can integrate into cellular membranes to form chloride ion channels and may contribute to cellular growth regulation [51–53]. Gene expression analysis shows that CLIC3 is predominantly enriched in lung tissues, with moderate levels in muscle, adipose tissue, and heart, and lower levels in the kidney and liver.

### 4.1. CLIC3 in Cell Senescence and Lung Injury

Lung injury is characterized by lung alveolar damage and can result from direct insults such as bacterial or viral infections, or indirect causes like systemic inflammation. Severe lung injury may progress to acute respiratory distress syndrome (ARDS) with approximately 40% of mortality rate [54]. Cellular senescence plays a significant role in the development of ARDS.

CLIC3 levels were significantly downregulated in rat lung tissues following formaldehyde (FA) inhalation exposure, suggesting a potential role of CLIC3 in FA-induced pulmonary damage [55]. Intratracheal bleomycin challenge is a widely used murine model of lung injury and pulmonary fibrosis. Lung injury was triggered by bleomycin in the first week, followed by fibroblast activation and differentiation to myofibroblast and elevated fibrotic responses. Luan, C. et al. reported elevated CLIC3 levels in lung tissues after eight days of bleomycin challenge [52]. The contrasting expression patterns of CLIC3 observed in FA and bleomycin-induced lung injury models reflect distinct molecular mechanisms underlying the regulation of CLIC3 expression during the different lung injury models. The modulation of CLIC3 levels in other experimental lung injury models remains to be investigated in future studies. CLIC3 is highly expressed in most lung cell types, with the exception of macrophages. Further studies using single-cell analysis or immunohistochemistry could help identify the specific cell types that alter CLIC3 expression in different murine models of lung injury.

Luan, C. et al. reported elevated CLIC3 in the lungs of aging mice compared to young mice [52], suggesting a potential role for CLIC3 in the aging process. Using bleomycin to induce cellular senescence in lung fibroblasts, the authors demonstrated that CLIC3 knockdown attenuated bleomycin-induced cellular senescence, suggesting that CLIC3 plays a pivotal role in promoting cellular senescence in the lungs, particularly within lung fibroblasts [52]. The authors investigated the molecular mechanisms underlying CLIC3’s role in cellular senescence. They showed that CLIC3 knockdown alleviated bleomycin treatment-induced chloride depletion and mitochondrial dysfunction in lung fibroblasts [52]. However, the connection between chloride regulation and cellular senescence remains insufficiently characterized. Additionally, the study revealed that CLIC3 negatively regulates and physically interacts with ERK7 [52], leading to the conclusion that ERK7 mediates the pro-cell senescence effects of CLIC3. The role of the CLIC3/ERK7 axis in the development of lung injury needs to be investigated in future studies.

### 4.2. CLIC3 in Lung Development and Cell Carcinoma

Transcriptomic analysis of human lung development identified CLIC3 within a principal component enriched by genes critical to early lung development [56], suggesting that CLIC3 may be part of a core regulatory network driving essential and overlapping processes during early lung formation. However, no study to date have demonstrated impaired lung development in CLIC3 knockout mice. Additional co-factors may be required for CLIC3 to exert its role in lung development.

Lung cancer may originate, in part, from the aberrant reactivation of developmental pathways that are typically active during embryogenesis. Luan, C. et al. analyzed CLIC3 expression in lung cancer and revealed a significant negative correlation between CLIC3 levels and lung cancer patient survival [52]. Moreover, they observed elevated CLIC3 expression in lung cancer cell lines compared to normal lung epithelial cells [52]. Beyond its established roles in chloride regulation, cellular senescence, and mitochondrial dysfunction, further research is needed to elucidate the impact of CLIC3 on lung cancer cell proliferation and metastasis.

## 5. CLIC4 in Lung Diseases

CLIC4 was originally identified as p64H1 (or HuH1) in rat tissues in 1997 [57], which is localized in the cytoplasm, intracellular organelle membrane, and plasma membrane. In addition to acting as a chloride channel, CLIC4 also play roles as an adaptor protein to regulate cytoskeletal rearrangement and cell division [58,59]. Relocation of CLIC4 between different cellular compartments contributes to the functions of CLIC4. CLIC4 is broadly expressed across various lung cell types; however, most studies have primarily focused on its role in the lung vascular endothelium.

### 5.1. CLIC4 in Pulmonary Arterial Hypertension (PAH)

#### 5.1.1. CLIC4 Expression in PAH

PAH is a chronic and progressive cardiopulmonary disease characterized by elevated pulmonary arterial pressure, ultimately leading to right ventricular failure. Pulmonary arterial endothelial cell dysfunction, including barrier disruption, resistance to apoptosis, and endothelial-to-mesenchymal transition (EndoMT), triggers proliferation and contraction of vascular smooth muscle cells. Contributing factors such as reduced nitric oxide (NO), Egln1, and increased inflammation have been shown to contribute to play key roles in driving endothelial cell dysfunction [60–62]. Proteomic analysis of lung homogenates from patients with PAH and control subjects revealed a significant upregulation of CLIC4 in PAH lungs [63]. This finding was validated by both immunoblotting and immunohistochemical staining. Notably, CLIC4 was prominently localized in the endothelium of remodeled pulmonary arteries in PAH lung tissues [63,64], highlighting its potential involvement in PAH-associated vascular remodeling. Wojciak-Stothard, B. et al. reported elevated CLIC4 in plasma and blood-derived endothelial cells from patients with PAH [64]. They also demonstrated that upregulation of CLIC4 in the pulmonary arterial endothelium across three distinct rat models of PAH [64]. Notably, genetic depletion of CLIC4 attenuated disease severity in a chronic hypoxia-induced PAH mouse model [64]. Collectively, these human and animal studies strongly support that increased CLIC4 levels in pulmonary arterial endothelium contribute to the pathogenesis of PAH.

#### 5.1.2. Molecular Mechanisms of CLIC4 Contribution to the Development of PAH

To further elucidate the molecular mechanisms by which CLIC4 contributes to the development of PAH, Wojciak-Stothard, B. et al. focused on investigating the role of CLIC4 in modulating endothelial cell function. Their findings demonstrated that CLIC4 enhances endothelial cell angiogenesis while impairing endothelial barrier integrity [64]. Subsequent studies from the same group further revealed that CLIC4 regulates several key signaling pathways involved in endothelial dysfunction and development of PAH, including activation of NF-κB, stabilization of hypoxia-inducible factor-1α, increased production of VEGF and endothelin-1, and mitochondrial dysfunction [65,66]. Additionally, CLIC4 was shown to promote neutrophil extracellular traps (NETs)-induced spheroid sprouting [67] and reduce BMPRII levels [66]. Importantly, the small GTPase Arf6 was identified as a regulator of CLIC4 function, further linking it to endothelial dysfunction and vascular remodeling in PAH [66]. The finding regarding the role of CLIC4 in lung endothelial cell function has been supported by independent studies using other endothelial cell lines. Notably, Kleinjan, M.L. et al. demonstrated that CLIC4 regulates the endothelial barrier in human umbilical vein endothelial cells (HUVECs) [68]. Collectively, these findings revealed multiple molecular mechanisms by which CLIC4 regulates endothelial cell function in the context of PAH. CLIC4 is ubiquitously expressed in lung cells, including vascular smooth muscle cells, which are central to the development of PAH. However, its specific role in the vascular smooth muscle cells remains unexplored. Wojciak-Stothard, B. et al. utilized whole-body CLIC4 knockout mice but did not distinguish the cell types in which CLIC4 exerts its primary effect [64]. Recently, endothelial-specific CLIC4 knockout mice have been developed, providing a valuable tool for investigating the role of CLIC4 in vascular biology [68]. Kleinjan, M.L. et al. demonstrated that these mice exhibit reduced lung vascular permeability in response to PAR1-activating peptide [68], highlighting the importance of CLIC4 in endothelial barrier regulation. The model offers a promising approach to elucidate the contribution of elevated CLIC4 in endothelial cells to the pathogenesis of PAH.

### 5.2. CLIC4 in Lung Regeneration and Tumorigenesis

Lung epithelial cells, including alveolar type 2 (AT2) cells, express CLIC4. AT2 cells serve as progenitors that different into alveolar type 1 (AT1) cells, a process essential for lung regeneration and the maintenance of alveolar barrier integrity following lung injury. Impaired progenitor function of AT2 cells can lead to unresolved lung injury, ultimately contributing to the development of lung fibrosis [69,70]. Single-cell RNA-seq analysis of AT2 to AT1 differentiation revealed a reduction of CLIC4 expression during the transition [71]. CLIC4 was shown to facilitate TGF-β signaling, a pathway critical for the trans-differentiation of AT2 to AT1 cells [71]. These findings suggest that CLIC4 may play a regulatory role in alveolar epithelial regeneration following lung injury. AT2-specific CLIC4 knockout mice have not yet been developed. Comparative studies using whole-body CLIC4 knockout, AT2-specific CLIC4 knockout, and endothelial-specific CLIC4 knockout mice in models of lung injury and fibrosis model could provide a comprehensive understanding of CLIC4’s role in lung epithelial and endothelial cells during lung inflammatory diseases. Moreover, it remains unclear whether CLIC4’s function is attributable to its chloride channel activity or its specific subcellular localization.

The role of CLIC4 in lung cancer has been reported by Okudela, K. et al. Five-year disease-free survival rate analysis revealed a positive correlation between CLIC4 levels and disease-free survival in stage I lung adenocarcinomas [72]. CLIC4 levels were reduced in KRAS V12-tranduced normal human bronchial epithelial cells (NHBECs), some lung cancer cell lines (A549, TKB14, and H2087), and primary lung cancer cells [72], suggesting that, unlike CLIC1 and CLIC3, CLIC4 may possess anti-tumor properties. Although detailed mechanistic studies were not conducted, the authors showed that CLIC4 downregulation in NHBECs enhanced clonogenicity and cellular proliferation [72]. The molecular mechanisms underlying the distinct roles of CLIC4, in contrast to CLIC1 and CLIC3, in lung cancer remain unclear. It would be valuable to investigate the subcellular translocation, chloride channel activity, and interacting partners of CLIC isoforms in lung cancer cells.

### 5.3. CLIC4 in Lung Inflammation

Intratracheal administration of lipopolysaccharide (LPS) is a widely used experimental model for inducing lung inflammation and injury. Luo, J. et al. reported a significant reduction in CLIC4 levels in lung tissues of mice challenged with LPS [73]. Complementary *in vitro* studies further suggest that CLIC4 exerts anti-inflammatory effects, evidenced by attenuating LPS-induced inflammatory responses in bronchial epithelial cells [73]. As previously discussed, CLIC4 regulates NF-κB activation and induces mitochondrial dysfunction in lung endothelial cells [66]. However, in lung epithelial cells exposed to LPS, CLIC4-mediated anti-inflammatory responses appear to occur independently of NF-κB activation and mitochondrial dysfunction. The mechanisms underlying these contrasting roles of CLIC4 in inflammatory signaling between lung endothelial and epithelial cells remain unclear. In this study, the authors revealed that CLIC4 overexpression mitigated LPS-induced reduction in intracellular chloride levels, which may account for the anti-inflammatory effects of CLIC4 in lung epithelial cells [73]. In contrast to these findings, He, G. et al. reported elevated CLIC4 levels in lungs and other organs, such as spleen, kidney, and brain, of mice following intraperitoneal LPS injection. Furthermore, CLIC4 knockout mice exhibited reduced systemic inflammation and improved survival rate [74], suggesting that CLIC4 may play a pro-inflammatory property in response to LPS. Although the latter study did not employ the intratracheal LPS-induced lung injury model, intraperitoneal LPS injection is also known to induce lung inflammation. Intratracheal administration of LPS primarily induces direct damage and inflammation to the lung epithelial cells, whereas intraperitoneal LPS triggers indirect lung injury through systemic inflammation that initially affects lung endothelial cells. As previously discussed, CLIC4 promotes pro-inflammatory signaling in lung endothelial cells but exhibits anti-inflammatory effects in lung epithelial cells. These cell type-specific functions may account for the contrasting roles of CLIC4 observed in the two distinct lung injury models.

## 6. Other CLICs in Lung Diseases

The role of CLIC2 in tumorigenesis and metastasis has been previously reported. However, to our knowledge, its role in the lungs has not been investigated, despite its ubiquitous expression across various lung cell types. CLIC5 has been reported to be downregulated in lung tumors, with lower expression levels negatively correlating with overall survival in lung adenocarcinoma patients [75,76]. However, the molecular mechanisms underlying CLIC5 downregulation and its contribution to lung tumorigenesis remain poorly understood. CLIC6 is predominantly expressed in the lungs and brain, and its chloride channel activity has been detected in mouse lung epithelial cells [77]. Nevertheless, the functional role of CLIC6 in lung epithelial biology is still largely unexplored. Abnormal chloride levels resulting from CFTR dysfunction play a critical role in the pathogenesis of CF. Increased CLIC6 levels at the plasma membrane may help regulate chloride levels and potentially mitigate the severity of CF.

## 7. Summary, Prospective Therapeutic Approaches, and Future Research Directions

CLIC proteins show distinct expression patterns across lung cell types. CLIC1, CLIC3, CLIC4, and CLIC5 have been implicated in lung carcinoma. However, molecular mechanisms by which CLIC proteins regulate tumor cell proliferation, migration, and cell death remain poorly understood. Future studies should determine whether membrane-bound or soluble forms play a role in lung tumorigenesis. In addition, CLIC1, CLIC3, and CLIC5 have been associated with lung injury and inflammation, yet key questions persist to be investigated, such as whether these isoforms form heterodimers and which specific cell types they influence. Among the family, CLIC4 in lung endothelial cells is well characterized; it regulates endothelial remodeling and barrier function in PAH and experimental lung injury.

IAA94 (R(+)-[(6,7-Dichloro-2-cyclopentyl-2,3-dihydro-2-methyl-1-oxo-1H-inden-5-yl)-oxy]acetic acid), an indanyloxyacetic acid derivative, has been widely employed as a pharmacological inhibitor of CLICs across various disease models. IAA-94 binds directly to CLIC proteins and reduces the frequency of channel opening. Notably, IAA94 has demonstrated efficacy in reducing pro-inflammatory cytokine levels in the liver of mice subjected to intraperitoneal LPS-induced sepsis [78], delaying vaso-occlusion in a mouse model of photochemical thrombus formation [79], increasing myocardial infarction [80], and suppressing food intake in mice. Given the involvement of CLICs in lung pathophysiology, particularly in inflammatory and vascular diseases, IAA94 may present a promising therapeutic strategy for lung disorders.

However, the contrasting roles of specific CLIC isoforms in lung epithelial versus endothelial cells highlight the need for cell-specific approaches. The development of lung cell-specific CLIC-deficient mouse models would enhance our understanding of isoform-specific functions and help identify precise therapeutic targets. Furthermore, targeted delivery of IAA94 via conjugation to cell specific markers could improve therapeutic efficacy while minimizing off-target effects. Notably, CLIC5 and CLIC6 possess distinct N-terminal structural features, which may enable the development of novel isoform-specific inhibitors that selectively target CLIC5 and CLIC6 without affecting other CLIC family members. The regulation of gene expression and protein stability of CLICs remains poorly understood. Elucidating the molecular mechanisms governing the regulation of CLICs may uncover novel therapeutic strategies for the treatment of lung disorders.
